# The Therapy of Vulvar Carcinoma—Evaluation of Surgical Options in a Retrospective Monocentric Study

**DOI:** 10.3390/life13101973

**Published:** 2023-09-27

**Authors:** Peter Jankowski, Sebastian Findeklee, Mihai-Teodor Georgescu, Romina Marina Sima, Meletios P. Nigdelis, Erich-Franz Solomayer, Gilbert Georg Klamminger, Bashar Haj Hamoud

**Affiliations:** 1 Department for Gynecology, Obstetrics and Reproductive Medicine, Saarland University Hospital, 66421 Homburg, Germany; 2 Department of Oncology, “Carol Davila” University of Medicine and Pharmacy, 050474 Bucharest, Romania; mihai.georgescu@umfcd.ro; 3 “Prof. Dr. Alexandru Trestioreanu” Oncology Institute, 022328 Bucharest, Romania; 4 Department of Obstetrics and Gynecology, “Carol Davila” University of Medicine and Pharmacy, 020021 Bucharest, Romania; romina.sima@umfcd.ro; 5 The “Bucur” Maternity, ‘Saint John’ Hospital, 040294 Bucharest, Romania; 6 Unit of Reproductive Endocrinology, 1st Department of Obstetrics and Gynecology, Papageorgiou General Hospital, Aristotle University of Thessaloniki, 56403 Thessaloniki, Greece

**Keywords:** vulvar cancer, surgical oncology, vulvectomy, hemi-vulvectomy, hemivulvectomy, lymphadenectomy, sentinel lymph node biopsy

## Abstract

(1) Background: Surgical-oncological treatment methods are continuously put to the test in times of evidence-based medicine—notably, a constant reevaluation remains key, especially for tumor entities with increasing incidence such as vulvar carcinoma. (2) Methods: In order to determine the postoperative clinical course of different methods of vulvar excision (vulvectomy, hemivulvectomy) as well as inguinal lymph node removal (lymphadenectomy, sentinel lymph node biopsy) with regard to postoperative wound-healingprocess, perioperative hemorrhage, and re-resection rates, we retrospectively analyzed surgical, morphological and laboratory data of 76 patients with a pathological diagnosed vulvar cancer. (3) Results: Analysis of our data from a single center revealed a comparable perioperative clinical course regardless of the chosen method of vulvar excision and inguinal lymph node removal. (4) Conclusions: Thus, our results emphasize the current multimodality in surgical therapy of vulvar carcinoma, in which consideration of known prognostic factors together with the individual patient’s clinical situation allow guideline-based therapy aimed at maximizing surgical safety.

## 1. Introduction

Vulvar carcinomas, although relatively uncommon in women, accounting for approximately only 4% of all female genital tract tumors, are experiencing an increase in incidence rates due to their multifactorial genesis and etiology, involving various known risk factors such as human papillomavirus (HPV) infection, vulvar dysplasia, use of immunosuppressants, inflammatory diseases of the genital area, and improved life expectancy, among others [1,2,3]. As a result, the available therapeutic options for vulvar carcinoma are constantly being reviewed and reassessed, with the aim of expanding or adapting them as necessary.

The treatment approach for vulvar carcinoma and its precursor lesions remains highly individualized and takes into account several factors. These include the size of the primary tumor, its extent of lymphatic spread, the presence of coexisting medical conditions in the patient, and, importantly, the anticipated cosmetic and functional outcomes, as well as the overall quality of life following treatment [4].

The primary goal of treating vulvar carcinoma is to achieve effective tumor control while minimizing the impact on the patient’s physical and psychological well-being. The final choice of treatment modality is influenced by patient, clinical, as well as pathological criteria, whereas the stage of the disease is subsequently assigned considering not only the spread of local invasion but also the presence or absence of regional lymph node metastasis. For early-stage vulvar carcinomas, localized surgical excision is often the treatment of choice, aiming to achieve complete tumor removal while preserving as much healthy tissue as possible. In cases where lymph node involvement is suspected or confirmed, a sentinel lymph node biopsy or complete lymphadenectomy may be performed to assess the spread of cancer cells and guide further treatment decisions [4].

In more advanced cases of vulvar carcinoma, a multimodal approach combining surgery, radiation therapy, and chemotherapy may be employed. This approach aims to maximize the chances of tumor control and improve overall survival rates. Surgery may involve radical excision of the tumor, often accompanied by the removal of adjacent lymph nodes. Radiation therapy, either external beam or brachytherapy, can be used to target residual tumor cells or as primary treatment in cases where surgery is not feasible. Chemotherapy, typically in the form of systemic administration, may be used in combination with surgery and/or radiation therapy to enhance treatment effectiveness and reduce the risk of disease recurrence [5].

The selection of the most appropriate therapeutic approach is a complex decision-making process that requires a multidisciplinary team, including gynecologic oncologists, radiation oncologists, medical oncologists, and pathologists. The team considers the individual patient’s characteristics, including age, overall health status, and personal preferences, in addition to the specific tumor characteristics. Treatment planning also involves discussions with the patient regarding potential short-term and long-term side effects, as well as the impact on body image, sexual function, and psychological well-being [6].

In recent years, efforts have been made to improve the quality of life for patients undergoing treatment for vulvar carcinoma. This includes the development of less invasive surgical techniques, such as minimally invasive and robotic-assisted procedures, which aim to minimize postoperative complications and improve cosmetic outcomes. Additionally, supportive care measures, including pain management, psychosocial support, and rehabilitation services, play a crucial role in addressing the holistic needs of patients and promoting their overall well-being throughout the treatment journey [7].

Whenever feasible, maximal surgical removal of tumor cells with a tumor-cell-free resection margin of ≥3 mm is first-line treatment in Germany [8]. Depending on the extent of excision, a distinction is made between partial/total vulvectomy as well as superficial/deep vulvectomy in dependence on the depth of resection. Since the risk of inguinal lymph node metastasis increases in accordance with the respective tumor thickness, vascular affection and histomorphological grading, lymphadenectomy (superficial inguinofemoral/deep femoral) is another crucial aspect of the therapy for patients with TNM stage ≥ pT1b [8,9]. Following the possible mode of singular lymph node extirpation in breast cancer and due to a high concomitant morbidity of bilateral inguinal lymphadenectomy, sentinel lymph node biopsy (SLNB) after technetium labeling was developed for vulvar carcinoma; actual indications for SLNB are, for example: stage carcinomas <4 cm, unifocal tumor occurrence, interdisciplinary experience, as well as clinically and sonographically unremarkable lymph nodes [8].

In the presented study, we retrospectively analyzed the postoperative course of surgical therapy options (hemivulvectomy/vulvectomy; inguinal lymphonodectomy/SLNB) as well as associated clinical parameters (e.g., postoperative wound-healing affections, rate of local recurrences) of patients with a histopathologically determined diagnosis of vulvar cancer. As validation-based research, our study aims to contribute to existing but clinically very important knowledge to allow the best evidence-based surgical decision making in patients with vulvar cancer. Analyzing surgical, morphological and laboratory data, our data thus emphasize once more the value of evidence-based oncologic therapy that is guided by the patient’s individual clinical course as well as established prognostic factors.

## 2. Materials and Methods

**Figure 1:**
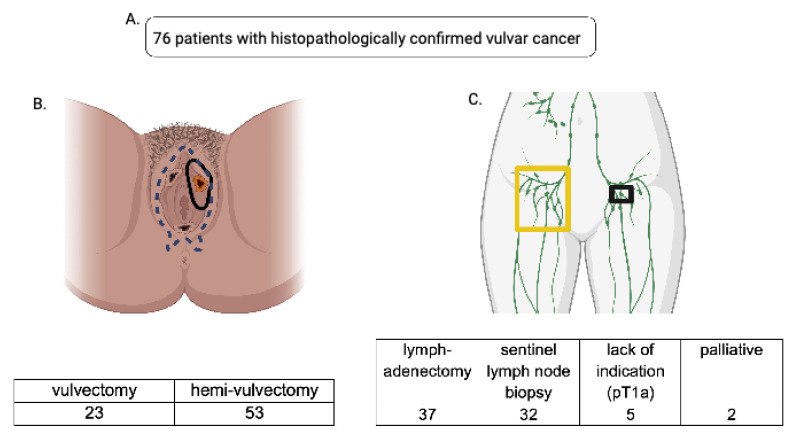
(**A**): A total of 76 patients who underwent primary surgery for vulvar carcinoma at the Clinic for Gynecology, Obstetrics and Reproductive Medicine (Saarland University Hospital) between 2006 and 2011 were included in our data analysis. (**B**): Data were split into two cohorts based on the radicality of surgical therapy; one cohort underwent vulvectomy (blue dotted line represents the extent of the resection), the other hemivulvectomy (black circular lining shows the extent of the resection). (**C**): Accordingly, the data were split with regard to the lymph node removal viz. lymphadenectomy (yellow-framed) and sentinel lymph node biopsy (black-framed)—7 patients were excluded due to their palliative status or lack of indication for lymph node removal.

In total, 92 patients were treated for vulvar carcinoma, melanoma of the vulva and Paget’s disease between 2006 and 2011 at the Clinic for Gynecology, Obstetrics and Reproductive Medicine (Saarland University Hospital). A total of 76 patients who underwent primary surgical therapy for (primary) squamous epithelial vulvar cancer between 2006 and 2011 (Supplementary Table S1) comprise the cohort analyzed in this study. From 2006–2011, 59 patients remained recurrence-free, and 17 developed tumor recurrences (histopathologically determined occurrence of invasive tumor cells). Exclusion criteria for the study were incomplete clinical data sets, singular noninvasive precancerous lesions such as Paget’s disease, melanoma of the vulva, and termination of therapy (discontinuation of guideline-compliant therapy). The study protocol was conducted in line with all requirements of the Ethics Committee of the Saarland for retrospective data collection and analysis; data are processed in line with the “EU General Data Protection Regulation” (2018) [10] and the world medical declaration (WMA) Declaration of Helsinki [11]. Patient follow-up was carried out according to the institutional protocol every 3 months in the first 3 years, then every 6 months in the following 2 years. The follow-up procedure included anamnesis, inguinal and cervical clinical examination, speculum examination, rectovaginal examination, and cytological pap smear examination. Regional pelvic ultrasound was used every 6 months for follow-up. HPV-testing and colposcopy were procedures optionally prescribed at follow-up. Initially, four distinct groups were defined in dependence of the oncological surgery performed. The first cohort underwent hemivulvectomy and the second cohort total vulvectomy as surgical treatment. In addition, a second distribution was performed, and patients were split once more based on the performed surgery of inguinal lymph node removal (sentinel lymph node biopsy (SLNB)/lymphadenectomy); see Figure 1 for a comprehensive overview. Clinically relevant perioperative parameters and tumor characteristics such as grading, histology, tumor size, FIGO stage, recurrence rates, postoperative wound-healing affections (dehiscence, infection), perioperative hemorrhage, as well as post-resection rates were collected and documented across all subgroups.

### Clinical Workflow and Data Analysis

In order to minimize postoperative wound-healing impairments, preoperative disinfection was performed in accordance with standard hygiene regulations. After the vulvar skin was disinfected with Octenisept^®^ (octenidine hydrochloride 0.1%, 2-phenoxyethanol 2%; Schülke & Mayer GmbH, Norderstedt, Germany) colposcopical examination of vulvar lesions could be performed presurgically. Dermal disinfection of the mid- and lower abdomen as well as inner thigh was carried out using Braunol^®^ (povidone-iodine, iodine, povidone; B. Braun Melsungen AG, Melsungen, Germany). All postoperative wound-healing affections were recorded and documented during the regular postoperative follow-up (id est, at least every three months within the first three years [8]). In order to address and quantify intraoperative hemorrhage, hemoglobin levels of all patients were evaluated before surgery and after surgery (24 h). All patients who underwent additional major or intermediate surgical performances such as hysterectomy and colporrhaphy were excluded, whereas patients with small additional procedures such as conization and fractional curettage were not excluded due to minimal associated bleeding risks. Overall, a total of 44 patients could be analyzed for relevant bleeding.

Whenever SLNB was performed, 150–200 MBq (megabecquerel) of Technetium 99 m-Nanocoll was injected subcutaneously next to the tumorous area 24 h presurgery. The quality of the procedure was tested after a latency period of 2 h, and all sentinel lymph node(s) as well as their explicit localization were visualized and directly marked using a CT/SPECT system (BrightView, Phillips^©^, Amsterdam, The Netherlands and Hawkeye, General Electrics^©^, Boston, MA, USA). Intraoperatively, 2 mL of Patent Blue V 25 mg/mL (active ingredient: Patent Blue V; manufacturer: Guerbet, Villepinte, France) was administered peritumorally after disinfection of the surgical area. Accordingly, intraoperative sentinel lymph node detection could be performed in a visually and auditorily controlled manner using a RMD Navigator GPS™ System (probes R3687 and R2847).

Statistics were performed using SPSS 15.0 (SPSS Inc., Chicago, IL, USA; version 2006). To capture the distribution of quantitative characteristics, arithmetic mean, median, standard deviation, as well as minimum and maximum values were calculated. Hypothesis testing was carried out using *t*-test for mean comparison and Fisher’s exact test for comparison of distribution of variables. Presumed intervariable relationships between two associated variables were ranked and described using Spearman’s rank correlation. In all cases, a probability of error of *p* ≤ 0.05 was assumed to be significant, and all *p* ≤ 0.01 were assumed to be highly significant.

## 3. Results

### 3.1. Defining the Cohort

**Figure 2:**
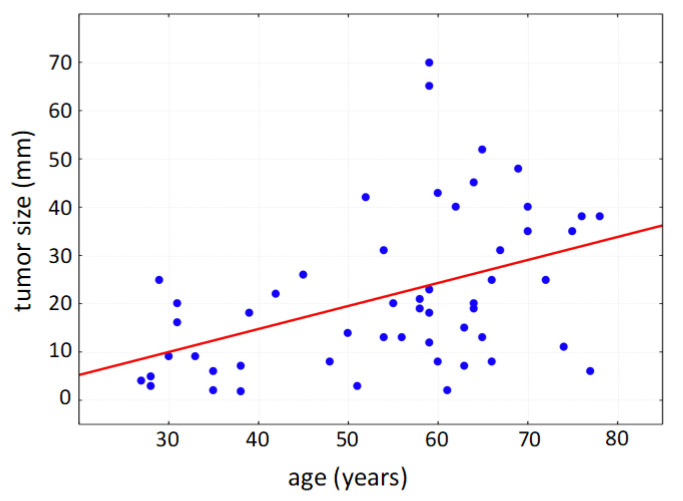
Positive correlation of tumor size with patient age (Spearman’s rank correlation, *p* < 0.001).

Within the collected data set of 76 patients undergoing surgery due to primary vulvar carcinoma, the mean age of the patients was 62 years (standard deviation: 16 years); the youngest patient was 28 years old; the oldest patient was 87 years old. Due to the multifocal tumor cell occurrence even in early stages, multifocal localization of vulvar carcinoma was diagnosed in 60% at the time of initial diagnosis (multiple counting possible). The vulvar carcinoma was localized frequently in the area of the clitoris (64%), hereby commonly affecting parts of the labia minora et majora pudendi. The urethra was tumor-affected in 18% of cases, and the posterior commissure was involved in 28%. As a tumor entity with defined precancerous lesions, vulvar intraepithelial neoplasia (VIN) occurred in 28 cases in addition to the diagnosed vulvar carcinoma; 27 of the affected patients showed histomorphological signs of a high-grade dysplasia (VHSIL/dVIN). The average size of the tumor was 25.1 mm in patients with affected lymph nodes and 19.9 mm in patients with unaffected lymph nodes, respectively; thereby, the tumor size correlated positively with patient age (Figure 2).

### 3.2. Vulvectomy, Hemivulvectomy, Sentinel Lymph Node Biopsy and Radical Lymphadenectomy—Surgical Procedures Put to the Test

A comparative analysis of previously defined cohorts (patients with vulvectomy/patients with hemivulvectomy) showed no increased risk for intraoperative or postoperative hemorrhage depending on the surgical method performed (Supplementary Table S2). In addition, evaluation of the re-resection rates showed no statistically significant difference when comparing their frequency in accordance to the respective initial surgical technique (*p* = 0.275). Within our patient population, no significant effect (*p* = 0.079) of the surgical method itself on the number of postoperative wound-healing affections could be determined; they occurred with a frequency of 26.1% in the vulvectomy group and a frequency of 9.4% in the hemivulvectomy group. Although the number of local recurrences was higher after hemivulvectomy in comparison to vulvectomy, the surgical method per se had no statistically significant effect (*p* = 0.743) on the local recurrence rate (Table 1). In the case of R1 resection, if the tumor cells microscopically attach to the resection margin, a local resection of residual tumor tissue was performed in a histologically controlled manner (R0); nevertheless, our analysis showed that patients in need of re-resection due to R1 status (*n* = 7) after first surgery were at higher risk of developing local recurrences (*p* < 0.001) and did develop recurrences rather at an earlier point (7.5 months vs. 12.25 months, *p* < 0.05) in comparison to patients with initial R0 resection.

SNLB and lymphadenectomy are per definition distinguishable by the amount of resected lymphoid tissue; a definition that reproducibly served our analysis as a quality control of the respective cohorts viz. patients with SNLB and patients with lymphadenectomy; see Supplementary Table S3 for detailed information. Although the percentage of affected lymph nodes is higher in primary lymphadenectomy than in SLNB, a direct comparison of the two surgical methods showed no significant difference in the surgical removal rate/detection rate of affected lymph nodes (*p* = 0.171). Likewise, there was no statistically significant difference in intra- and postoperative hemorrhages between both groups (*p* = 0.739).

## 4. Discussion

From 2006–2011, 70% of all vulvar carcinomas presenting to our clinic were treated with a hemivulvectomy; solely 30% of tumors required a total vulvectomy, a formation in line with the recent development towards a preferably organ-preserving therapy [12,13]. Terminologically, the clinically used term hemivulvectomy is further specified, inter alia, as anterior-, anterior right-, anterior left-, isolated right-, isolated left-, and posterior-hemivulvectomy. Like the width of resection, the depth of surgical tissue removal can be further classified, using the periosteum of the pubis and the superficial aponeurosis of the urogenital diaphragm as landmarks for deep excision. Whilst this concept warrants inter-study comparability and would be justified in a larger cohort, we renounced adopting it in order to aim for balanced data sets.

One of the pioneering studies in the field of surgical management of vulvar carcinoma was conducted by Homesley et al. [14]. This study aimed to assess the effectiveness of radical vulvectomy, a surgical procedure involving the complete removal of the vulva, in the treatment of vulvar carcinoma. The results of the study provided valuable insights into the survival outcomes associated with this surgical approach. In the study, Homesley and colleagues evaluated a cohort of patients with vulvar carcinoma, stratified based on the stage of the disease. The 5-year survival rates were calculated for patients with both early-stage and advanced-stage disease. The findings revealed that patients with early-stage disease who underwent radical vulvectomy achieved a 5-year survival rate of 60%. This suggests that radical vulvectomy can be an effective treatment option for patients with localized vulvar carcinoma, leading to favorable long-term survival outcomes. However, it is important to note that the study also indicated that patients with advanced-stage disease had a lower 5-year survival rate of 30% following radical vulvectomy. This highlights the challenges associated with advanced disease and the need for additional treatment modalities in these cases. For patients with advanced-stage vulvar carcinoma, a multimodal treatment approach, combining surgery with other therapeutic interventions such as chemotherapy and radiation therapy, may be necessary to achieve optimal outcomes.

The study by Homesley et al. [14] provided valuable insights into the survival rates associated with radical vulvectomy for vulvar carcinoma. However, it is essential to consider certain limitations of the study. The study design was retrospective, which may have introduced biases and limitations inherent to this type of analysis. Additionally, the study did not explore the impact of other variables, such as lymph node involvement, tumor size, or histological subtype, on survival outcomes.

Despite these limitations, the study by Homesley and colleagues [14] remains an important contribution to the field, as it shed light on the effectiveness of radical vulvectomy as a surgical treatment option for vulvar carcinoma. Subsequent studies have built upon these findings and have explored various surgical techniques and treatment approaches to further optimize outcomes for patients with vulvar carcinoma.

In cases involving en bloc resection, particularly with a butterfly skin incision, extended postoperative morbidities such as necrosis and infections have been documented. However, the use of the triple-incision method has shown promise in reducing these complications by minimizing wound areas and preserving healthy tissue [15]. Given that vulvectomy entails a larger surgical area compared to hemivulvectomy, the likelihood of encountering postoperative wound management complications is inherently higher. To address these challenges, a study conducted by Dargent et al. [16] evaluated the effectiveness of conservative surgery, which involves the selective removal of only the affected tissue, in combination with adjuvant radiotherapy. The results of the study demonstrated a 5-year survival rate of 84% for patients with early-stage disease and 56% for patients with advanced disease when this approach was employed. The implementation of conservative surgery offers several advantages. By targeting only the affected tissue, the procedure minimizes the extent of tissue removal, thereby preserving healthy tissue and reducing the risk of complications. This approach is particularly beneficial in cases where the tumor is localized and has not spread extensively. Adjuvant radiotherapy serves as an additional treatment modality, helping to eradicate any remaining cancer cells and further improve the chances of long-term survival. Furthermore, the use of conservative surgery allows for better preservation of the cosmetic and functional aspects of the vulvar region. By minimizing tissue excision, the procedure aims to maintain the natural appearance and integrity of the vulva, leading to improved postoperative cosmetic outcomes. Additionally, preserving healthy tissue helps to maintain normal physiological functions, such as sexual and urinary function, leading to a better quality of life for patients [16]. While the study by Dargent and colleagues [16] demonstrates promising results, further research is necessary to validate and refine the use of conservative surgery in combination with adjuvant radiotherapy for vulvar carcinoma. Ongoing studies should focus on evaluating long-term survival rates, recurrence rates, and the impact of this approach on patient-reported outcomes such as quality of life and functional well-being.

In a study conducted by Piura et al. in 2013 [17], the outcomes of two surgical approaches for vulvar carcinoma, namely radical vulvectomy and skin-sparing vulvectomy, were compared. Radical vulvectomy involves the removal of the entire vulvar region, including the affected tissue and surrounding healthy skin. On the other hand, skin-sparing vulvectomy aims to selectively remove only the affected tissue while preserving as much of the surrounding healthy skin as possible. The study findings indicated that skin-sparing vulvectomy had several advantages over radical vulvectomy. Firstly, skin-sparing vulvectomy was associated with a lower risk of postoperative complications, particularly wound healing problems. By preserving a larger portion of healthy skin, the surgical site had improved healing capacity, reducing the likelihood of wound breakdown or delayed wound healing. This is of significant importance, as complications in wound healing can lead to increased morbidity, prolonged hospital stays, and additional interventions. Furthermore, skin-sparing vulvectomy demonstrated favorable cosmetic outcomes. By preserving a greater amount of healthy skin, the procedure aimed to maintain the natural appearance and contours of the vulva to a larger extent. This contributed to improved postoperative aesthetics and patient satisfaction. The preservation of healthy skin also played a role in minimizing functional impairments, such as discomfort during daily activities and sexual intercourse, which can arise from extensive tissue excision [17]. The study by Piura et al. [17] shed light on the potential benefits of skin-sparing vulvectomy in the management of vulvar carcinoma. However, it is important to note that the selection of the surgical approach should be based on individual patient characteristics, tumor stage, and the extent of involvement. In certain cases where the tumor is large or has infiltrated nearby structures, a radical vulvectomy may be necessary to achieve complete tumor removal and ensure adequate disease control. It is worth mentioning that the study by Piura et al. [17] represents an important contribution to the field, providing evidence for the feasibility and advantages of skin-sparing vulvectomy. However, further research and long-term studies are needed to validate these findings, assess oncologic outcomes, and evaluate the impact on long-term survival rates.

Yet our data did not support clear evidence of superiority of hemivulvectomy regarding this aspect; in this regard, factors such as proper oncological indication prior to surgery and accurate preoperative adherence to hygiene standards, as well as thorough postoperative follow-up, are essential. In our patient cohort, the number of local recurrences was not affected by the surgical method per se; therefore, hemivulvectomy as well as vulvectomy are regarded as equivalent with regard to the respective oncological outcome. Instead, the surgical therapy of choice should consider the individual oncological situation, aiming not only for R0 resection but a resection margin including a tumor-cell-free rim [18,19]. Looking at our data, the number of local recurrences (23%) is in line with the literature [20]. Since patients with an initial R1 resection required a follow-up resection more frequently—their recurrences occurred three times faster than in the women with initial R0 resection prior to recurrence—intraoperative frozen sections could thereby help in assessing local tumor infiltration zones directly with an accuracy rate up to 98.6% [21].

Since not only lymphatic inguinal metastasis but also the extent of it [22] and even lymphatic vessel infiltration [23] per se are prognostically relevant, adequate surgical removal as well as the histomorphology examination of the inguinal lymph nodes remain important factors. On the one hand, oncological safety must be ensured; on the other hand, concomitant morbidity should be reduced. Since radical lymphadenectomy is efficient—its groin recurrence rate is 1% to 10%—there is no need to perform it in patients without lymph node metastasis (65–75%) [24]. Le T. and colleagues in 2004 [25] evaluated the effectiveness of SLNB and found that this approach had a diagnostic accuracy of 95% and reduced the risk of complications such as lymphedema and wound infection compared to traditional lymphadenectomy. Also, Klapdor and colleagues in 2018 [26] evaluated the outcomes of different methods of inguinal lymph node removal, including open lymphadenectomy and minimally invasive techniques such as laparoscopic and robotic-assisted lymphadenectomy. The study found that minimally invasive techniques resulted in a shorter hospital stay and lower risk of complications compared to open lymphadenectomy. Therefore, SLNB provides an outstanding alternative and should always be considered with regard to the individual patient and tumor in case of suspected lymph node metastasis [27]. A completely different perspective was proposed in a recent study by Preti et al. [28], who studied how the involvement of inguinal lymph nodes changed over a period of ten years in patients over 40 years old who had been diagnosed with vulvar squamous cell carcinoma through histological examination. In total, the authors screened 760 patients for lymph node involvement, lymph node dissection and number of positive lymph nodes. After all, they did not determine a progress over time in detecting lymph node involvement and its extent at an earlier time point of disease progression. Within our cohort, a median of two lymph nodes were removed when SNLB was performed, while during lymphadenectomy an average of six to seven lymph nodes were removed. In summary, a total of 23% of the patients with lymph node removal had a histologically proven metastasis within our analysis, whereas Preti et al. determined a lymph node involvement of 39.7% in the 1980s and of 48.5% in the 2010s.

Postoperative radiotherapy of the groin area aiming at recurrency prevention in patients with positive nodal status (≥2 lymph node metastases, >5 mm, tumor growth exceeding the fibrous capsule) is a key factor in adjuvant therapy [8,29,30]. Forward-thinking future studies will evaluate the use and advantage of radical lymphadenectomy in individual situations of proven inguinal lymphatic metastasis [31], potentially similar to axillar lymph node affects and their subsequent therapy options in mammary cancer [32].

## 5. Conclusions

In the current medical literature, there is a growing body of evidence supporting the use of various surgical options for the treatment of vulvar carcinoma. Each surgical approach has its own set of advantages and limitations, and the choice of technique should be carefully considered based on individual patient factors and tumor characteristics.

To contribute to already existing knowledge on surgical management modalities of vulvar carcinoma, we conducted a retrospective study involving 76 patients who underwent primary vulvar surgery for previously diagnosed vulvar cancer. The study aimed to analyze anamnestic and clinical data to evaluate the postoperative clinical course of different surgical techniques. By assessing the clinical course and outcomes of patients undergoing different surgical approaches, we aimed to provide valuable insights into the effectiveness and safety of these methods.

Our data analysis revealed that the clinical course of patients was comparable regardless of the chosen method of vulvar excision and inguinal lymph node removal. This finding suggests that the selection of a specific surgical approach did not significantly influence the overall clinical outcomes in our cohort. However, it is important to note that the study design was retrospective, which may introduce certain limitations and potential biases in the analysis. The relatively small sample size may restrict the detection of subtle differences between groups and increase the risk of a Type II error, as not all variables were statistically analyzed. The definition of groups and details regarding surgical procedures, pathological information and adjuvant therapies were not fully described, limiting a comprehensive understanding of the disease characteristics and outcomes. Future studies with larger cohorts and more comprehensive data collection are encouraged to validate and expand upon our findings. When determining the type of surgical resection for vulvar carcinoma, it is crucial to consider well-known oncological and biological risk factors. These factors include the stage of the disease, the size and location of the tumor, the presence of lymph node metastasis, and other prognostic indicators. By incorporating these factors into the surgical decision-making process, healthcare professionals can strive to achieve maximum oncological safety and optimize treatment outcomes.

As our study contributes to existing knowledge on the surgical treatment of vulvar cancer, it is embedded in such a theoretical framework. However, such existing conditions not only confirm the clinical relevance of our study but also help to assess the value and utility of our findings in the clinical context—the latter always resulting from the individual multicenter experiences of different research groups addressing the same question/topic. In the context of this validation-based research task, our study contributes to existing but highly clinically relevant knowledge. Still, it remains essential to acknowledge the need for further research in this field. Larger-scale prospective trials are needed to compare different surgical techniques, assess long-term outcomes, and evaluate the impact on survival rates, recurrence rates, and quality of life. Moreover, future studies should also explore advancements in minimally invasive surgical approaches and the role of adjuvant therapies in enhancing treatment outcomes.

In conclusion, our study adds to the growing body of literature on the surgical management of vulvar carcinoma. We observed comparable clinical outcomes among patients undergoing different surgical approaches, emphasizing the importance of individualized treatment decisions based on well-established oncological and biological risk factors. However, further research, particularly larger prospective trials, is warranted to validate our findings and advance our understanding of the optimal surgical management of vulvar carcinoma. By continuing to explore and refine surgical techniques, we can improve patient outcomes and strive for the best possible care for individuals affected by vulvar carcinoma.

## Figures and Tables

**Table 1:** Within our patient collective, the surgical method (vulvectomy/hemivulvectomy) per se showed no statistically significant disadvantage (*p* = ns) regarding re-resection rates, postoperative wound-healing affections or local recurrency rates. Results are shown in absolute numbers and percentages; *p*-value was calculated using *t*-test.

	Hemivulvectomy (*n* = 53)	Vulvectomy (*n* = 23)	*p*
re-resection rates	12 (22.6%)	8 (34.8%)	0.275
wound-healing affections	5 (9.4%)	6 (26.1%)	0.079
local recurrency	10 (19%)	3 (14%)	0.743

## Data Availability

Please contact the corresponding author for individual arrangements.

## Supplementary Materials

The following supporting information can be downloaded at: https://www.mdpi.com/article/10.3390/life13101973/s1, Supplementary Table S1—Number of patients (absolute numbers and percentages) treated by total vulvectomy and hemivulvectomy in the years 2006–2011; Table S2—Mean decrease in hemoglobin level after surgery in patients who underwent primary vulvar cancer surgery in 2006–2011. ns = not significant; Table S3—Number of histologically determined lymph nodes removed during SNB and lymphonodectomy.

## References

[1] Joura E.A., Giuliano A.R., Iversen O.-E., Bouchard C., Mao C., Mehlsen J., Moreira E.D., Ngan Y., Petersen L.K., Lazcano-Ponce E. (2014). A 9-valent HPV vaccine against infection and intraepithelial neoplasia in women. N. Engl. J. Med..

[2] Madeleine M.M., Finch J.L., Lynch C.F., Goodman M.T., Engels E.A. (2016). HPV-related cancers after solid organ transplantation in the United States. Am. J. Transpl..

[3] Saraiya M., Unger E.R., Thompson T.D., Lynch C.F., Hernandez B.Y., Lyu C.W., Steinau M., Watson M., Wilkinson E.J., Hopenhayn C. (2020). US assessment of HPV types in cancers: Implications for current and 9-valent HPV vaccines. J. Natl. Cancer Inst..

[4] Hacker N.F., Berek J.S., Juillard G.J., Lagasse L.D. (1984). Preoperative radiation therapy for locally advanced vulvar cancer. Cancer.

[5] Urban R., Chen L.-M., Karlan B.Y., Bristow R.E., Li A.J. (2015). Perioperative and Critical Care. Gynecologic Oncology: Clinical Practice and Surgical Atlas.

[6] National Comprehensive Cancer Network (NCCN) Vulvar Cancer (Version 3.2021). https://www.nccn.org/professionals/physician_gls/pdf/vulvar.pdf.

[7] Nandwani M., Barmon D., Begum D., Liegise H., Kataki A.C. (2019). An Overview of Vulvar Cancer: A Single-Center Study from Northeast India. J. Obstet. Gynaecol. India.

[8] Diagnosis, Therapy and Follow-Up Care of Vulvar Cancerand Its Precursors. National Guideline of the German Society of Gynecology and Obstetrics (S2k-Level, AWMF Registry No. 015/059, August 2015). http://www.awmf.org/leitlinien/detail/ll/015-059.html.

[9] Sedlis A., Homesley H., Bundy B.N., Marshall R., Yordan E., Hacker N., Lee J.H., Whitney C. (1987). Positive Groin Lymph Nodes in Superficial Squamous Cell Vulvar Cancer. A Gynecologic Oncology Group Study. Am. J. Obstet. Gynecol..

[10] The European General Data Protection Regulation (GDPR). https://eur-lex.europa.eu/EN/legal-content/summary/general-data-protection-regulation-gdpr.html.

[11] The World Medical Association (1964). WMA Declaration of Helsinki—Ethical Principles for Medical Research Involving Human Subjects. [abgerufen am 09. November 2020]. https://www.wma.net/policies-post/wma-declaration-of-helsinki-ethical-principles-for-medical-research-involving-human-subjects/.

[12] Giannini A., D’Oria O., Chiofalo B., Bruno V., Baiocco E., Mancini E., Mancari R., Vincenzoni C., Cutillo G., Vizza E. (2022). The Giant Steps in Surgical Downsizing toward a Personalized Treatment of Vulvar Cancer. J. Obstet. Gynaecol. Res..

[13] Dellinger T.H., Hakim A.A., Lee S.J., Wakabayashi M.T., Morgan R.J., Han E.S. (2017). Surgical Management of Vulvar Cancer. J. Natl. Compr. Cancer Netw..

[14] Homesley H.D., Bundy B.N., Sedlis A., Yordan E., Berek J.S., Jahshan A., Mortel R. (1983). Assessment of current International Federation of Gynecology and Obstetrics staging of vulvar carcinoma relative to prognostic factors for survival (a Gynecologic Oncology Group study). Am. J. Obstet. Gynecol..

[15] Siller B.S., Alvarez R.D., Conner W.D., McCullough C.H., Kilgore L.C., Partridge E.E., Austin J.M. (1995). T2/3 Vulva Cancer: A Case-Control Study of Triple Incision versus En Bloc Radical Vulvectomy and Inguinal Lymphadenectomy. Gynecol. Oncol..

[16] Dargent D., Martin X., Sacchetoni A., Mathevet P. (2000). Laparoscopic assessment of the sentinel lymph nodes in early vulvar cancer. Gynecol. Oncol..

[17] Piura B., Rabinovich A., Leron E., Yanai-Inbar I. (2013). Skin-sparing vulvectomy vs. radical vulvectomy in the treatment of vulvar carcinoma: A matched-pair comparison of surgical and oncological outcomes. Gynecol. Oncol..

[18] Rodolakis A., Diakomanolis E., Voulgaris Z., Akrivos T., Vlachos G., Michalas S. (2000). Squamous Vulvar Cancer: A Clinically Based Individualization of Treatment. Gynecol. Oncol..

[19] Chan J.K., Sugiyama V., Pham H., Gu M., Rutgers J., Osann K., Cheung M.K., Berman M.L., Disaia P.J. (2007). Margin Distance and Other Clinico-Pathologic Prognostic Factors in Vulvar Carcinoma: A Multivariate Analysis. Gynecol. Oncol..

[20] Yap J.K.W., O’Neill D., Nagenthiran S., Dawson C.W., Luesley D.M. (2017). Current Insights into the Aetiology, Pathobiology, and Management of Local Disease Recurrence in Squamous Cell Carcinoma of the Vulva. BJOG Int. J. Obstet. Gynaecol..

[21] Horn L.-C., Wagner S. (2010). Frozen Section Analysis of Vulvectomy Specimens: Results of a 5-Year Study Period. Int. J. Gynecol. Pathol. Off. J. Int. Soc. Gynecol. Pathol..

[22] van der Velden J., van Lindert A.C., Lammes F.B., ten Kate F.J., Sie-Go D.M., Oosting H., Heintz A.P. (1995). Extracapsular Growth of Lymph Node Metastases in Squamous Cell Carcinoma of the Vulva. The Impact on Recurrence and Survival. Cancer.

[23] Yoder B.J., Rufforny I., Massoll N.A., Wilkinson E.J. (2008). Stage IA Vulvar Squamous Cell Carcinoma: An Analysis of Tumor Invasive Characteristics and Risk. Am. J. Surg. Pathol..

[24] Zivanovic O., Khoury-Collado F., Abu-Rustum N.R., Gemignani M.L. (2009). Sentinel Lymph Node Biopsy in the Management of Vulvar Carcinoma, Cervical Cancer, and Endometrial Cancer. Oncologist.

[25] Covens A., Vella E.T., Kennedy E.B., Reade C.J., Jimenez W., Le T. (2015). Sentinel lymph node biopsy in vulvar cancer: Systematic review, meta-analysis and guideline recommendations. Gynecol Oncol..

[26] Desimone C.P., Elder J., van Nagell J.R. (2011). Selective inguinal lymphadenectomy in the treatment of invasive squamous cell carcinoma of the vulva. Int J Surg Oncol..

[27] Oonk M.H.M., van Os M.A., de Bock G.H., de Hullu J.A., Ansink A.C., van der Zee A.G.J. (2009). A Comparison of Quality of Life between Vulvar Cancer Patients after Sentinel Lymph Node Procedure Only and Inguinofemoral Lymphadenectomy. Gynecol. Oncol..

[28] Preti M., Bucchi L., Micheletti L., Privitera S., Corazza M., Cosma S., Gallio N., Borghi A., Bevilacqua F., Benedetto C. (2021). Four-decade trends in lymph node status of patients with vulvar squamous cell carcinoma in northern Italy. Sci. Rep..

[29] Deppe G., Mert I., Winer I.S. (2014). Management of Squamous Cell Vulvar Cancer: A Review. J. Obstet. Gynaecol. Res..

[30] Matak L., Dukić B., Tupek T., Lisica-Šikić N., Mikuš M. (2020). Primary ectopic lobular breast cancer of the vulva: Case report and review of literature. J. Obstet. Gynaecol..

[31] Woelber L., Mahner S., Voß C., Trillsch F. (2020). The Role of Lymphadenectomy in Vulvar Cancer: Current Standards and Future Directions. Cancers.

[32] Galimberti V., Cole B.F., Zurrida S., Viale G., Luini A., Veronesi P., Baratella P., Chifu C., Sargenti M., Intra M. (2013). Axillary Dissection versus No Axillary Dissection in Patients with Sentinel-Node Micrometastases (IBCSG 23-01): A Phase 3 Randomised Controlled Trial. Lancet Oncol..

