# Supplementary material for: The Therapy of Vulvar Carcinoma—Evaluation of Surgical Options in a Retrospective Monocentric Study
Source: Life (Basel). 2023 Sep 27;13(10):1973. doi: 10.3390/life13101973 (PMC10608767; doi:10.3390/life13101973)
Supplement: Supplementary file 1 [file life-13-01973-s001.zip › life-2427534-supplementary.pdf]

SUPPLEMENTARY MATERIAL - The Therapy of Vulvar Carcinoma - Evaluation of Surgical Options in a Retrospective Monocentric Study

Table S1. Number of patients (absolute numbers and percentages) treated by total vulvectomy and hemivulvectomy in the years 2006-2011.

	2006 n=13	2007 n=7	2008 n=11	2009 n=12	2010 n=16	2011 n=17	Total n=76
vulvectomy	4<br>(30,7%)	5<br>(71,4%)	1<br>(9%)	5<br>(41,6%)	5<br>(31,3%)	3 1<br>(7,6%)	23<br>(30,2%)
hemivulvectomy	9<br>(69,3%)	2<br>(28,6%)	10<br>(91%)	7 5<br>(8%)	11<br>(68,7%)	14<br>(82,4%)	53<br>(69,8%)

Table S2. Mean decrease in hemoglobin level after surgery in patients who underwent primary vulvar cancer surgery in 2006-2011. ns = not significant

	hemivulvectomy (n=32)	vulvectomy (n=12)	*p*
drop in hemoglobin levels postoperative (g/dL)	1,4±0,8 (+SLNB)<br>1,7±1,4(+lymphadenectomy)	1,7±1,2 (+SLNB)<br>1,7±1,2 (+lymphadenectomy)	*ns*

Table S3. Number of histologically determined lymph nodes removed during SNB and lymphonodectomy.

	Average number of lymph nodes removed ± SD
SLNB - right side	1,96±1,62
SLNB - left side	2,37±1,58
right lymphadenectomy	6,34±2,92
left lymphadenectomy	6,79±3,12
unilateral lymphadenectomy	8,13±5,89
